# Unequal access in a digital age: women's digital exclusion and socioeconomic inequalities in Vietnam

**DOI:** 10.3389/fdata.2025.1718366

**Published:** 2025-12-04

**Authors:** Chi Thi Lan Pham, Quyen Thi Tu Bui, Anh Ha Le, Long Quynh Khuong

**Affiliations:** 1 Faculty of Sciences, University of British Columbia, Vancouver, BC, Canada; 2 Department of Biostatistics, Hanoi University of Public Health, Hanoi, Vietnam; 3 College of Arts and Sciences, University of Pennsylvania, Philadelphia, PA, United States; 4 Department of Biostatistics, Epidemiology, and Informatics, Perelman School of Medicine, University of Pennsylvania, Philadelphia, PA, United States

**Keywords:** ICT4D, digital exclusion, digital skills, women, Vietnam, MICS 2021, SDGs

## Abstract

**Introduction:**

Access to information and communication technologies (ICTs) and the skills to use them are essential for inclusive development and digital participation. As Vietnam accelerates its digital transformation, ensuring that women are not left behind is critical to achieving the Sustainable Development Goals (SDGs), particularly SDG 5 (Gender Equality) and SDG 9 (Industry, Innovation, and Infrastructure). This study investigates the extent and socioeconomic patterning of digital exclusion among women in Vietnam.

**Methods:**

We utilized nationally representative data from the 2021 Multiple Indicator Cluster Survey (MICS), which covered 10,770 women aged 15–49. Digital exclusion was defined in terms of (1) no ICT access (no use of computer, internet, or mobile phone in the past 3 months) and (2) no ICT skills (unable to perform any of nine standard digital tasks).

**Results:**

Results show that 4.28% of women lacked digital access and 72.85% lacked digital skills. Inequalities were stark: access was lowest among ethnic minorities (19.55%) and the poorest quintile (17.10%), compared to 1.980.31% in the majority and richest groups. The digital skills gap was even wider, with 95.51% of the poorest women lacking ICT skills vs. 41.23% of the richest. Multivariable logistic regressions confirmed that ethnicity, wealth, rural residence, and older age were key predictors of exclusion.

**Conclusion:**

These findings underscore the urgent need for inclusive digital policies that extend beyond infrastructure to address gendered and socioeconomic barriers to digital literacy. Without targeted efforts, digital rollouts may widen existing inequalities and undermine SDG progress.

## Introduction

The rapid expansion of information and communication technologies (ICTs) has reshaped economies, education, and societies worldwide (Sarkar, 2012). ICTs are no longer optional tools for communication but have become essential enablers of participation in the labor market, lifelong learning, and civic engagement (Sarkar, 2012; Mavrou et al., 2017). The Sustainable Development Goals (SDGs) acknowledge this centrality by monitoring both access (Indicator 17.8.1) and skills (Indicator 4.4.1; United Nations, 2023). While mobile phone ownership and internet penetration have grown rapidly, many women, particularly in remote areas, still face barriers to accessing these technologies. Even when connectivity is available, access does not always translate into digital competencies (GSMA, 2021; GDIP, 2024). From a capability perspective, digital participation depends not only on physical access but also on individuals' real opportunities and skills to use technology effectively This dual challenge risks widening socioeconomic disparities, as individuals lacking access and/or skills are excluded from opportunities in education, health, and employment (Helsper, 2012; Law et al., 2018; OECD, 2019).

Vietnam provides a valuable case study of these broader dynamics. The country is undergoing a rapid digital transformation, where access to ICT and skills are increasingly becoming prerequisites for daily life. Essential services such as the national electronic identification system (VNeID), digital health insurance, online tax declaration, and e-business platforms now require citizens to engage digitally (Government of the Socialist Republic of Vietnam, 2024). Yet persistent inequalities by education, wealth, ethnicity, and place of residence may prevent some groups, particularly women, from benefiting equally from this transition. Applying Amartya Sen's Capability Approach helps explain how such structural inequalities constrain women's ability to convert digital access into meaningful use, resulting in capability deprivation even when infrastructure exists. Addressing these gaps is critical not only for Vietnam but also for other LMICs pursuing digitalization as part of their development strategies.

Despite growing attention, two evidence gaps remain. First, most studies report access (e.g., phone or internet use) or skills in isolation; few construct clearly interpretable indicators that identify those with no digital access or no ICT skills, limiting the policy's ability to see who is completely disconnected vs. who is connected but unskilled (Law et al., 2018; OECD, 2019). Second, even when such indicators are available, analyses seldom systematically map their distribution across social and economic strata to pinpoint which groups are most at risk of exclusion in the digital era, and thus where interventions should be targeted. This study aims to use nationally representative data from Vietnam's 2021 MICS6, which builds on MICS5 ICT access/use items by introducing standardized ICT skills aligned with SDG 4.4.1 (General Statistics Office UNICEF, 2021) to address these gaps. Guided by the capability approach, we conceptualize digital exclusion as the deprivation of capabilities shaped by socioeconomic and demographic inequalities. First, we construct two policy-relevant indicators, “no digital access” and “no ICT skills”, to distinguish complete disconnection from capability deprivation. Second, we map the distribution of each indicator across education, wealth, ethnicity, urban–rural residence, and region to identify the most digitally hard-to-reach women and those without ICT skills, thereby informing targets for intervention and providing a baseline for monitoring progress toward equitable digital inclusion in Vietnam and comparable LMICs.

## Methodology

### Study sample

The MICS is a nationally representative household survey program developed by UNICEF to provide internationally comparable data on children and women (General Statistics Office UNICEF, 2021). Surveys employ a multistage, stratified cluster sampling design to ensure representativeness across urban and rural areas and the six geographic regions of Vietnam. In 2021, among the interviewed households, 11,294 women aged 15–49 years were identified; 10,770 were successfully interviewed, yielding a response rate of 95.4% among eligible women. This analysis uses the women's questionnaire dataset from MICS 2021 (MICS6).

### Study measurements

#### Study outcome

No digital access: we defined “no digital access” as reporting no use of each of the following in the preceding 3 months: a computer or tablet, the internet, and a mobile phone. Respondents who answered “not at all” to all three items were coded as having no digital access.

No ICT skills: we defined “no ICT skills” as reporting none of nine ICT-related tasks in the preceding 3 months: copying/moving a file; using copy-paste in a document; sending an e-mail with an attachment; using arithmetic formulas in a spreadsheet; connecting and installing a new device; installing and configuring software; creating an electronic presentation; transferring a file between devices; and writing a computer program (MICS items MT6A–MT6I). Respondents who reported zero tasks were coded as having no ICT skills.

#### Independent variables

Key sociodemographic characteristics were included as independent variables. Age was grouped into standard five-year intervals (15–19, 20–24, 25–29, 30–34, 35–39, 40–44, and 45–49 years). Ethnicity was classified into two categories: Kinh/Hoa (the majority population) and non-Kinh (all other ethnic minority groups) to ensure sufficient sample sizes for analysis. Socioeconomic status was measured using the household wealth index, constructed through principal component analysis of household assets and housing characteristics. This index is standardized in the MICS (General Statistics Office UNICEF, 2021) and other large household surveys in low- and middle-income countries and has been validated in Vietnam (Vu et al., 2011). The index was divided into quintiles, ranging from the poorest (Q1) to the richest (Q5). Region of residence followed the six national geographic areas used in the MICS sampling design: Red River Delta, Northern Midlands and Mountains, North Central and Central Coastal, Central Highlands, Southeast, and Mekong River Delta. Place of residence was categorized as urban or rural, consistent with the national sampling frame and administrative classifications of the General Statistics Office. Migration status was defined based on self-reported change of residence. Women who reported changing their living location in the last 5 years were classified as migrants ( ≤ 5 years), while those who had not moved during that period were classified as non-migrants (>5 years).

### Statistical analysis

We estimated survey-weighted prevalences and 95% confidence intervals (CIs) for no digital access and no ICT skills, overall and by key subgroups (ethnicity, wealth quintile, region, and urban/rural residence). All estimates incorporate individual weights, clustering, and stratification to reflect the complex survey design.

To examine adjusted associations, we fitted two multivariable logistic regression models with outcomes: (1) no digital access in the past 3 months, and (2) no ICT skills. Models included the covariates listed above. We report odds ratios (ORs**)** with 95% CIs; *p* < 0.05 denotes statistical significance. Analyses were conducted in Stata 18 using the svy command.

### Ethical approval

This study utilized de-identified, publicly available data from the 2021 Vietnam Multiple Indicator Cluster Survey (MICS). MICS tools and protocols follow standard ethical review procedures; this secondary analysis of de-identified public data did not require additional review.

## Results

### Characteristics of the study sample

Table 1 summarizes the sociodemographic profile of the 10,770 women in the MICS sample. Nearly two-thirds identified as Kinh/Hoa (64.3%), while 35.7% belonged to other ethnic groups. Most were currently married/in union (76.9%), and the majority lived in rural areas (68.3%). Educational attainment varied: 11.5% had no formal education, 29.5% completed lower secondary education, 26.1% completed upper secondary education, and 18.1% held a university/college degree or higher. Wealth was distributed across quintiles (from 32.8% in Q1 to 15.5% in Q5). Regarding migration, 15.1% of respondents had changed their residence within the last 5 years.

**Table 1:** Characteristics of the study sample (MICS Vietnam 2021, women; *n* = 10,770).

**Factor**	**Category**	***n* (%)**
Sample size		11,294
Age group (years)	15–19	1,349 (12.5)
	20–24	1,150 (10.7)
	25–29	1,603 (14.9)
	30–34	1,797 (16.7)
	35–39	1,819 (16.9)
	40–44	1,635 (15.2)
	45–49	1,417 (13.2)
Ethnicity	Kinh or Hoa	7,261 (64.3)
	Other ethnic groups	4,033 (35.7)
Marital status	Currently married/in union	8,681 (76.9)
	Formerly married/in union	587 (5.2)
	Never married/in union	2,008 (17.8)
	Missing/don't know	7 (0.1)
Place of residence	Urban	3,573 (31.7)
	Rural	7,710 (68.3)
Region	Red River Delta	2,064 (18.3)
	Northern Midlands and Mountain	2,431 (21.5)
	North Central and Central Coastal	1,426 (12.6)
	Central highlands	1,334 (11.8)
	South east	2,300 (20.4)
	Mekong River Delta	1,728 (15.3)
Education	Pre-primary or none	1,234 (11.5)
	Primary	1,592 (14.8)
	Lower secondary	3,181 (29.5)
	Upper secondary	2,461 (22.9)
	Vocational high school	354 (3.3)
	University/college or higher	1,947 (18.1)
	Missing/don't know	1 (<0.1)
Wealth index quintile	Q1 (poorest)	3,707 (32.8)
	Q2	1,911 (16.9)
	Q3	1,726 (15.3)
	Q4	1,671 (14.8)
	Q5 (richest)	1,755 (15.5)
	No information	524 (4.6)
Migration status (past 5 years)	No migration	9,588 (84.9)
	Migrated ≤ 5 years	1,706 (15.1)

### Patterns of digital access and skills

Table 2 summarizes recent digital access and ICT skills among women aged 15–49 years. Approximately one in five reported using a computer or tablet at least once a week in the past 3 months. Internet use was common, with a majority reporting almost daily use. Mobile phone use was also widespread, with roughly seven in ten reporting any use in the prior 3 months. Engagement in ICT skills was comparatively limited, with about one in six respondents reporting basic tasks, including copying/moving files (16.9%), copying and pasting (16.6%), and emailing with attachments (16.8%). Fewer reported using spreadsheets (15.5%), installing/configuring software (13.0%), or creating presentations (7.9%). Advanced skills were rare (programming 1.4%).

**Table 2:** Distribution of digital access and ICT skills among women aged 15–49 years old, Vietnam MICS 2021 (*n* = 10,770).

**Factor**	**Category**	***n* (%)**
Computer/tablet usage during the last 3 months	Not at all	8,270 (76.8)
	Less than once a week	229 (2.1)
	At least once a week	563 (5.2)
	Almost every day	1,700 (15.8)
Internet usage during the last 3 months	Not at all	1,300 (12.1)
	Less than once a week	154 (1.4)
	At least once a week	904 (8.4)
	Almost every day	8,411 (78.1)
	No response	1 (<1)
Mobile phone usage during the last 3 months	Not at all	3,266 (30.3)
	Less than once a week	228 (2.1)
	At least once a week	918 (8.5)
	Almost every day	6,352 (59.0)
	No response	1 (<1)
Able to copy or move a file/folder		1,818 (16.9)
Able to send an e-mail with an attached file		1,804 (16.8)
Able to use copy/paste in a document		1,791 (16.6)
Able to use arithmetic formula in a spreadsheet		1,673 (15.5)
Able to install and configure software		1,402 (13.0)
Able to connect and install a new device		1,126 (10.5)
Able to transfer a file		1,058 (9.8)
Able to create an electronic presentation		853 (7.9)
Able to write a computer program		149 (1.4)

### Prevalence of digital exclusion, no digital access, and no ICT skills, by sociodemographic group

Overall, 4.3% of women (95% CI: 3.7–4.9) reported having no digital access in the 3 months preceding the survey. Inequalities were most pronounced by ethnicity and household wealth. Among ethnic minority women, 19.6% (16.4–22.7) lacked access compared with 2.0% (1.6–2.4) among Kinh/Hoa women. Similarly, the prevalence declined sharply from 17.1% (14.6–19.6) in the poorest quintile (Q1) to 0.3% (0.0–0.6) in the wealthiest quintile (Q5). By residence, 6.2% (5.2–7.1) of rural women reported no access, compared to 1.1% (0.7–1.5) of urban women. Across regions, levels ranged from 1.1% (0.7–1.5) in the Southeast to 17.2% (12.8–21.5) in the Central Highlands.

**Figure 1:**
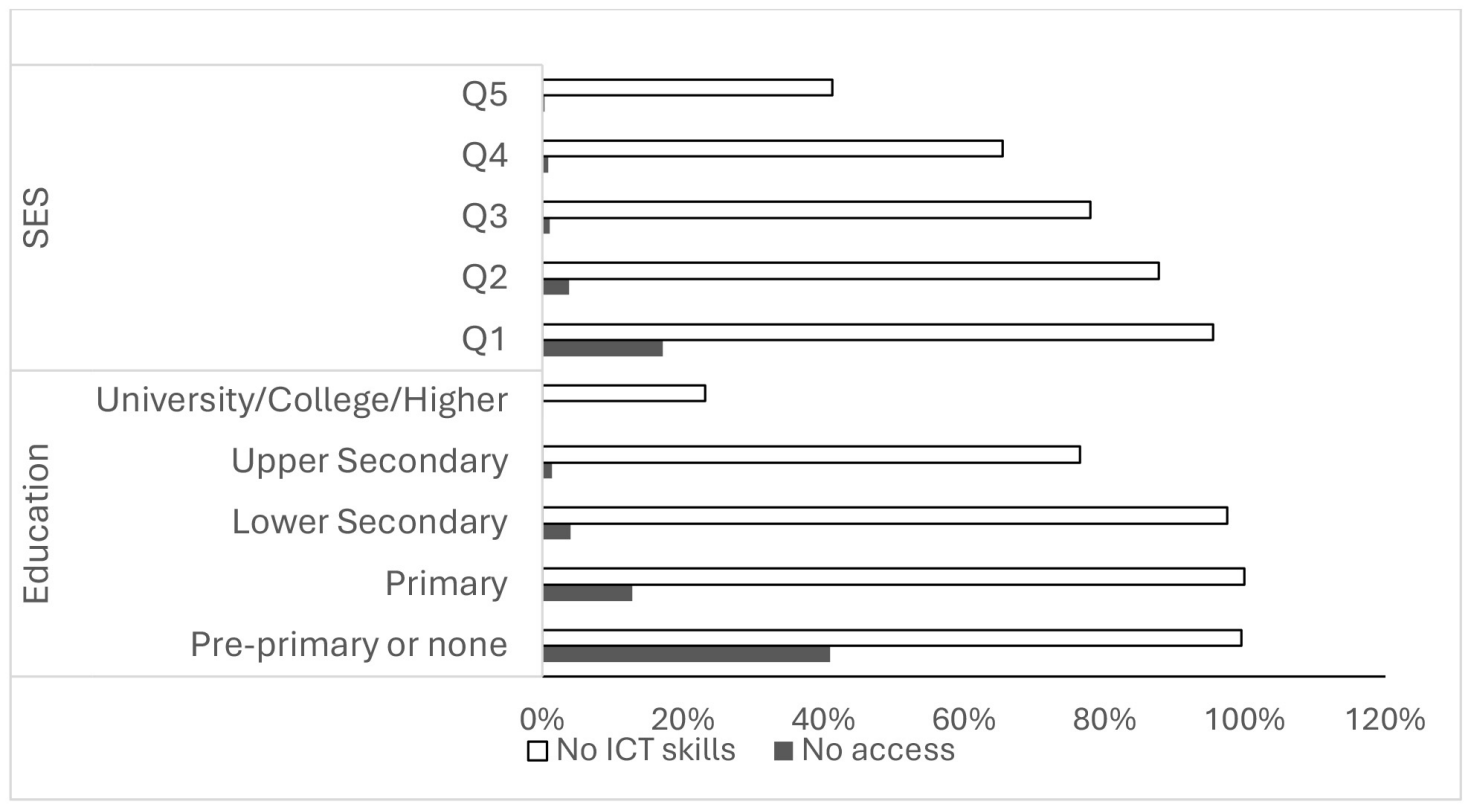
Inequalities in ICT access and ICT skills among women aged 15–49 years old, Vietnam MICS 2021 (*n* = 10,770) by SES and Education.

For ICT skills, the overall prevalence of women reporting no digital skills was 72.9% (70.9–74.7). A transparent socioeconomic gradient was observed, ranging from 95.5% (94.1–96.9) in Q1 to 41.2% (37.9–44.6) in Q5, with skill deficits being exceptionally high among women with low educational levels. By ethnicity, 92.5% (90.4–94.5) of minority women lacked ICT skills compared with 69.9% (67.8–72.0) among Kinh/Hoa. Rural-urban differences were also substantial, with 83.0% (81.3–84.7) in rural areas vs. 55.9% (52.4–59.5) in urban areas. Regionally, the highest proportions were found in the Northern Midlands and Mountain regions, as well as the Central Highlands (both around 83%), while the Southeast and Mekong River Delta recorded the lowest proportions (65.3 and 63.7%, respectively). Figure 1 illustrates inequalities by socioeconomic status and education, while Table 3 provides detailed prevalence estimates by demographic characteristics.

**Table 3:** Inequalities in ICT access and ICT skills among women aged 15–49 years old, Vietnam MICS 2021 by demographic characteristics (*n* = 10,770).

**Group**	**No ICT access prevalence (95%CI)**	**No ICT skills (%) prevalence (95%CI)**
Overall	4.28 (3.67; 4.90)	72.85 (70.95; 74.74)
**By ethnicity**		
Kinh/Hoa	1.98 (1.55; 2.41)	69.88 (67.80; 71.96)
Other ethnic groups	19.55 (16.44; 22.67)	92.47 (90.41; 94.54)
**By migration status**		
Non-migrants (≥5 years)	4.92 (4.19; 5.65)	75.64 (73.90; 77.37)
Migrated ≤ 5 years	1.55 (0.97; 2.12)	60.86 (56.01; 65.70)
**By residence**		
Urban	1.11 (0.72; 1.50)	55.92 (52.39; 59.46)
Rural	6.18 (5.22; 7.14)	82.97 (81.25; 84.68)
**By region**		
Red River Delta	2.05 (1.22; 2.88)	63.74 (59.57; 67.91)
Northern Midlands and Mountain	8.65 (5.56; 11.73)	83.33 (77.94; 88.72)
North Central and Central Coastal	4.45 (2.98; 5.92)	71.90 (66.60; 77.20)
Central highlands	17.18 (12.83; 21.52)	83.33 (79.70; 86.96)
South east	1.09 (0.66; 1.53)	65.30 (61.55; 69.05)
Mekong River Delta	3.69 (2.34; 5.04)	63.74 (59.57; 67.91)

### Multivariable correlates of digital exclusion: no digital access and no ICT skills

No Digital Access (Last 3 Months): as shown in Table 4, ethnicity, education, and wealth are the strongest correlates of this phenomenon. Compared with Kinh/Hoa, women from ethnic minority/other groups had more than twice the odds of no access (OR = 2.64; 95% CI: 2.07–3.38; *p* < 0.001). Odds declined steeply with wealth (vs. Q1: Q2 = 0.45 [0.35–0.59], Q3 = 0.23 [0.15–0.34], Q4 = 0.16 [0.09–0.30], Q5 = 0.08 [0.03–0.23]; all *p* ≤ 0.001). Education was strongly protective even at lower levels (vs. pre-primary/none: primary = 0.43 [0.35–0.52], lower secondary = 0.17 [0.14–0.21], upper secondary = 0.08 [0.06–0.11], university/college+ = 0.01 [0.00–0.04]; all *p* < 0.001). Regionally, the Central Highlands had higher odds (OR = 1.73; 1.14–2.62; *p* = 0.01), while the Southeast East (0.51; 0.31–0.84; *p* = 0.01), Mekong River Delta (0.50; 0.33–0.77; *p* < 0.001), and Northern Midlands and Mountains (0.49; 0.32–0.74; *p* < 0.001) had lower odds than the Red River Delta; the North Central and Central Coastal region did not differ. Rural residence trended higher but was not significant after adjustment (1.30; 0.97–1.73; *p* = 0.08). Interaction tests between ethnicity and wealth, as well as between ethnicity and education, showed no significant effects. The logistic regression model showed a good fit, with a pseudo-*R*^2^ of 0.35 and an area under the ROC curve of 0.95, indicating excellent discrimination between women with and without digital access.

**Table 4:** Logistic regression results for no access to ICT and no ICT skills among women aged 15–49 years old, Vietnam MICS 2021 (*n* = 10,770).

**Variable**	**No digital access OR (95% CI)**	***p*-value**	**No ICT skills OR (95% CI)**	***p*-value**
**Age (15–19 ref.)**				
20–24	0.90 (0.66–1.23)	0.52	3.89^**^ (2.97–5.09)	<0.001
25–29	0.67^*^ (0.50–0.91)	0.01	5.61^**^ (4.37–7.20)	<0.001
30–34	0.52^**^ (0.38–0.71)	<0.001	4.24^**^ (3.35–5.37)	<0.001
35–39	0.85 (0.63–1.15)	0.29	4.49^**^ (3.50–5.76)	<0.001
40–44	0.66^*^ (0.48–0.91)	0.01	5.00^**^ (3.72–6.71)	<0.001
45–49	0.85 (0.62–1.17)	0.32	7.52^**^ (5.42–10.45)	<0.001
**Education (pre-primary/none ref.)**				
Primary	0.43^**^ (0.35–0.52)	<0.001	2.11 (0.13–33.87)	0.60
Lower secondary	0.17^**^ (0.14–0.21)	<0.001	0.11^*^ (0.02–0.84)	0.03
Upper secondary	0.08^**^ (0.06–0.11)	<0.001	0.01^**^ (0.00–0.10)	<0.001
University/college+	0.01^**^ (0.00–0.04)	<0.001	0.00^**^ (0.00–0.01)	<0.001
**Ethnicity (Kinh/Hoa ref.)**				
Other/missing	2.64^**^ (2.07–3.38)	0.00	1.27 (0.98–1.64)	0.07
**Residence (urban ref.)**				
Rural	1.30 (0.97–1.73)	0.08	1.41^**^ (1.20–1.65)	<0.001
**Region (Red River Delta ref.)**				
Northern Midlands and Mountain	0.49^**^ (0.32–0.74)	<0.001	0.97 (0.73–1.29)	0.84
North Central and Central Coastal	1.17 (0.76–1.78)	0.48	1.03 (0.81–1.31)	0.82
Central highlands	1.73^**^ (1.14–2.62)	0.01	0.82 (0.62–1.08)	0.16
South east	0.51^**^ (0.31–0.84)	0.01	0.71^**^ (0.57–0.87)	<0.001
Mekong River Delta	0.50^**^ (0.33–0.77)	<0.001	0.74^*^ (0.57–0.96)	0.02
**Wealth quintile (Q1 ref.)**				
Q2	0.45^**^ (0.35–0.59)	<0.001	0.70^*^ (0.51–0.95)	0.02
Q3	0.23^**^ (0.15–0.34)	<0.001	0.51^**^ (0.37–0.70)	<0.001
Q4	0.16^**^ (0.09–0.30)	<0.001	0.33^**^ (0.24–0.45)	<0.001
Q5	0.08^**^ (0.03–0.23)	<0.001	0.20^**^ (0.14–0.28)	<0.001
**Migration (no migrated** ≤ **5 years ref.)**				
Migrated ≤ 5 years	0.88 (0.66–1.16)	0.35	1.03 (0.85–1.24)	0.77
Intercept	0.58 (0.32–1.06)	0.07	202.62^**^ (27.57–1489.38)	<0.001

^**^p < 0.01, ^*^p < 0.05.

No ICT Skills: wealth and education show strong, graded protection. Relative to the poorest quintile (Q1), odds of having no ICT skills fall monotonically across Q2–Q5 (Q2: OR = 0.70, 95% CI 0.51–0.95, *p* = 0.02; Q3: 0.51, 0.37–0.70, *p* < 0.001; Q4: 0.33, 0.24–0.45, *p* < 0.001; Q5: 0.20, 0.14–0.28, *p* < 0.001). Compared with pre-primary/none, lower secondary (OR = 0.11, 0.02–0.84, *p* = 0.03), upper secondary (0.01, 0.00–0.10, *p* < 0.001), and university/college+ (0.00, 0.00–0.01, *p* < 0.001) are associated with markedly lower odds. Older age strongly predicts no skills. Compared with the 15–19 age group, all older age bands have higher odds: 20–24 (3.89, 2.97–5.09), 25–29 (5.61, 4.37–7.20), 30–34 (4.24, 3.35–5.37), 35–39 (4.49, 3.50–5.76), 40–44 (5.00, 3.72–6.71), and 45–49 (7.52, 5.42–10.45); all *p* < 0.001. Rural residence is associated with higher odds (1.41, 1.20–1.65, *p* < 0.001). Ethnicity trends higher but is not significant after adjustment (minority/another vs. Kinh/Hoa: 1.27, 0.98–1.64, *p* = 0.07). Regionally, odds are lower in the Southeast (0.71, 0.57–0.87, *p* < 0.001) and Mekong River Delta (0.74, 0.57–0.96, *p* = 0.02); other regions do not differ from the Red River Delta. Recent migration ( ≤ 5 years) is not associated with a lack of skills (1.03, 0.85–1.24, *p* = 0.77). Interaction tests between ethnicity and wealth and between ethnicity and education showed no significant effects. In terms of model fit, the logistic regression model had a pseudo-*R*^2^ of 0.54 and an area under the ROC curve of 0.95, indicating strong predictive performance for identifying women without ICT skills.

## Discussion

Using nationally representative MICS6 (2021) data, this study provides an integrated view of women's digital exclusion in Vietnam by examining both no digital access and no ICT skills. We find that connectivity deprivation is relatively small, with 4.28% reporting no access in the prior 3 months. In contrast, capability deprivation is pervasive, affecting 72.85% of respondents who reported having no ICT skills. Although 4% may appear modest, scaled to the national population, it corresponds to approximately one million women without digital access, underscoring that connectivity expansion remains necessary even as capability emerges as the dominant constraint. Exclusion is highly stratified: odds of both outcomes fall monotonically with wealth and education; rural residence and ethnic minority status are associated with greater exclusion; odds of having no ICT skills increase with age; and regional disparities persist, with higher exclusion in the Central Highlands and lower exclusion in the Southeast and Mekong River Delta. Guided by the capability approach, these patterns reflect not only unequal access to resources but also unequal opportunities to convert access into meaningful digital participation shaped by social, educational, and structural constraints.

These patterns align with evidence from other LMICs, where device ownership and internet penetration have expanded faster than digital literacy, particularly among women and disadvantaged groups (GSMA, 2021; International Telecommunication Union, 2023). For example, Ghana and Bangladesh report high connectivity but low skill prevalence among women (Ghana Statistical Service, 2018; Bangladesh Bureau of Statistics, 2020). In Vietnam, this reflects a transition of the digital divide, from connectivity to capability, where infrastructure gaps are closing but skill gaps persist.

The magnitude of inequality is substantial: women with no schooling are over five times more likely to lack ICT skills than those with tertiary education, and ethnic minority women are twice as likely to be unskilled compared to Kinh women. The disadvantage of older cohorts likely stems from generational exposure gaps and limited digital socialization during their working lives. In contrast, younger women, although more connected, still face quality and relevance barriers in skill development. Regional inequalities may reflect structural factors, such as disparities in infrastructure investment, uneven education systems, and sociocultural norms that affect women's participation in digital spaces (OECD, 2019; GSMA, 2021; Ing et al., 2023).

These findings point to several policy-relevant considerations. Rather than emphasizing infrastructure alone, future rollouts (e.g., e-ID, digital health, e-tax, and social protection platforms) could be accompanied by basic digital skills support, helping ensure that connectivity translates into usable competence.

Overall, the results underscore that Vietnam's digital transformation cannot rely on connectivity expansion alone. True inclusion depends on building digital capabilities, ensuring that individuals have not only access to devices but also the competence to use them effectively. This shift from access to skills, from connectivity to capability, represents the next frontier of digital equity. For monitoring and SDG accountability, the two simple indicators used here, no digital access (SDG 9–aligned) and no ICT skills (SDG 4.4–aligned), could be reused in future MICS rounds to track who is being reached and where gaps are closing, contingent on maintaining consistent question wording, reference periods, and weighting.

These findings must be interpreted critically in the context of Vietnam's accelerated policy digitalization. The data show that capability deprivation is most profound among ethnic minorities, rural residents, and the poorest women. Consequently, “business-as-usual” digital rollouts, even if well-intentioned, are not neutral. Mandating digital engagement for essential services (such as e-ID or digital health) without parallel, equity-focused investments in foundational skills for these specific groups will likely result in a lack of inclusivity. Instead, such policies risk actively widening existing socioeconomic divides, creating new barriers to access, and deepening the very exclusion they aim to solve.

This study has limitations. ICT use and skills are self-reported, with a 3-month reference period, which may lead to misclassification of competence relative to performance-based assessments. Potential self-report and non-response bias could underestimate exclusion, as less connected women may be more challenging to reach or less confident in self-assessing their skills. The skills battery captures basic tasks but omits advanced competencies that are increasingly relevant to work and public service. As such, it reflects foundational rather than higher-order digital literacy. The cross-sectional design precludes causal inference, limiting claims of directionality or temporal change. Strengths include national representativeness, standardized measures, a large sample size enabling precise equity disaggregation, and a capability-plus-connectivity lens that advances measurement beyond connectivity alone and is transferable to other LMIC settings. For SDG monitoring, these findings demonstrate that combining both indicators provides a low-cost and interpretable framework for assessing digital inclusion equity under SDG 4.4 and 9.c. Finally, our analysis is limited to women aged 15–49, the standard MICS sample for these indicators. This scope does not allow for a direct comparison with men or an examination of digital exclusion among older populations (50+), who likely face distinct and potentially more severe capability gaps.

In summary, while Vietnam has essentially narrowed the access gap, capability remains the significant barrier: nearly three in four women lack ICT skills, and roughly one million still lack any access. Policies that integrate infrastructure with targeted, equity-focused skills investment and monitor progress using the two indicators are essential to ensure that digital transformation advances inclusion rather than deepening divides.

## Data Availability

The datasets presented in this study can be found in online repositories. The names of the repository/repositories and accession number(s) can be found below: https://microdata.worldbank.org/index.php/catalog/5956.
