# Supplementary material for: Unequal access in a digital age: women's digital exclusion and socioeconomic inequalities in Vietnam
Source: Front Big Data. 2025 Dec 4;8:1718366. doi: 10.3389/fdata.2025.1718366 (PMC12713119; doi:10.3389/fdata.2025.1718366)
Supplement: Supplementary file 1 [file Table_1.docx]

*Supplement table: Logistic regression results for no access to ICT and no ICT skills among Women Aged 15*–*49 years old, Viet Nam MICS 2021 (n=10,770) – with interaction terms*

| ***Variable*** | **No digital access** | | **No ICT skills** | |
| --- | --- | --- | --- | --- |
|  | **OR** | **p-value** | **OR** | **p-value** |
| **Age (15–19 ref.)** |  |  |  |  |
| 20–24 | 0.91 (0.66, 1.25) | 0.55 | 3.94 (3.00, 5.16) | 0.00 |
| 25–29 | 0.68 (0.50, 0.92) | 0.01 | 5.78 (4.49, 7.43) | 0.00 |
| 30–34 | 0.52 (0.38, 0.72) | 0.00 | 4.47 (3.52, 5.67) | 0.00 |
| 35–39 | 0.86 (0.64, 1.17) | 0.35 | 4.71 (3.67, 6.05) | 0.00 |
| 40–44 | 0.68 (0.49, 0.93) | 0.01 | 5.35 (3.98, 7.19) | 0.00 |
| 45–49 | 0.87 (0.63, 1.20) | 0.40 | 8.01 (5.77, 11.10) | 0.00 |
| **Education (Pre-primary/none ref.)** | | | | |
| Primary | 0.46 (0.24, 0.89) | 0.02 | 8.57 (0.53, 139.03) | 0.13 |
| Lower Secondary | 0.16 (0.08, 0.30) | 0.00 | 0.60 (0.08, 4.42) | 0.62 |
| Upper Secondary | 0.09 (0.05, 0.20) | 0.00 | 0.09 (0.01, 0.65) | 0.02 |
| University/College/Higher | 0.02 (0.00, 0.08) | 0.00 | 0.01 (0.00, 0.05) | 0.00 |
| **Ethnicity (Kinh/Hoa ref.)** |  |  |  |  |
| Other/Missing | 2.72 (1.47, 5.06) | 0.00 | 0.69 (0.46, 1.04) | 0.08 |
| ***Interactions*** |  |  |  |  |
| Pre-primary or none # Kinh or Hoa | 1 |  | 1 |  |
| Pre-primary or none # Other/Missing | 1 |  | 1 |  |
| Primary # Kinh or Hoa | 1 |  | 1 |  |
| Primary # Other/Missing | 0.91 (0.46, 1.80) | 0.79 | 1 |  |
| Lower Secondary # Kinh or Hoa | 1 |  | 1 |  |
| Lower Secondary # Other/Missing | 1.16 (0.58, 2.32) | 0.68 | 30.25 (4.02, 227.56) | 0.00 |
| Upper Secondary # Kinh or Hoa | 1 |  | 1 |  |
| Upper Secondary # Other/Missing | 0.76 (0.34, 1.69) | 0.50 | 2.10 (1.31, 3.36) | 0.00 |
| University/College/Higher # Kinh or Hoa | 1 |  | 1 |  |
| University/College/Higher # Other/Missing | 1 |  | 1 |  |
| Missing/DK # Kinh or Hoa | 1 |  | 1 |  |
| Missing/DK # Other/Missing | 1 |  | 1 |  |
| **Residence (Urban ref.)** |  |  |  |  |
| Rural | 1.31 (0.98, 1.75) | 0.07 | 1.44 (1.23, 1.68) | 0.00 |
| **Region (Red River Delta ref.)** |  |  |  |  |
| Northern Midlands & Mountain | 0.49 (0.32, 0.74) | 0.00 | 1.01 (0.75, 1.35) | 0.97 |
| North Central & Central Coastal | 1.16 (0.76, 1.78) | 0.49 | 1.03 (0.81, 1.30) | 0.84 |
| Central Highlands | 1.73 (1.14, 2.62) | 0.01 | 0.82 (0.62, 1.09) | 0.17 |
| Southeast | 0.51 (0.31, 0.84) | 0.01 | 0.71 (0.58, 0.87) | 0.00 |
| Mekong River Delta | 0.50 (0.32, 0.77) | 0.00 | 0.76 (0.58, 0.98) | 0.04 |
| **Migration (No migrated ≤5 yrs ref.)** |  |  |  |  |
| Migrated<=5 years | 0.88 (0.67, 1.16) | 0.36 | 1.02 (0.85, 1.23) | 0.81 |
| ***Wealth quintile (Q1 ref.)*** |  |  |  |  |
| Wealth index quintiles=2 | 0.45 (0.35, 0.59) | 0.00 | 0.77 (0.56, 1.07) | 0.12 |
| Wealth index quintiles=3 | 0.23 (0.15, 0.34) | 0.00 | 0.55 (0.40, 0.77) | 0.00 |
| Wealth index quintiles=4 | 0.16 (0.09, 0.29) | 0.00 | 0.35 (0.25, 0.48) | 0.00 |
| Wealth index quintiles=5 | 0.08 (0.03, 0.22) | 0.00 | 0.21 (0.15, 0.30) | 0.00 |
| **Intercept** | **0.55 (0.25, 1.23)** | **0.15** | **26.70 (3.57, 199.89)** | **0.00** |
